# Diphenyl (cyclo­pentyl­amido)­phospho­nate

**DOI:** 10.1107/S1600536811017028

**Published:** 2011-05-11

**Authors:** Fahimeh Sabbaghi, Mehrdad Pourayoubi, Poorya Zargaran, Giuseppe Bruno, Hadi Amiri Rudbari

**Affiliations:** a Department of Chemistry, Zanjan Branch, Islamic Azad University, PO Box 49195-467, Zanjan, Iran; b Department of Chemistry, Ferdowsi University of Mashhad, Mashhad 91779, Iran; c Dipartimento di Chimica Inorganica, Vill. S. Agata, Salita Sperone 31, Università di Messina, 98166 Messina, Italy

## Abstract

In the title mol­ecule, C_17_H_20_NO_3_P, the P atom is bonded in a distorted tetra­hedral environment. The dihedral angle between the two phenyl rings is 23.52 (10)°. The phosphoryl and N—H groups are *anti* with respect to one another. The –CH_2_–CH_2_–CH_2_–CH_2_– sequence of atoms in the cyclo­pentyl ring is disordered over two sets of sites with refined occupancies of 0.574 (10) and 0.426 (10). In the crystal, mol­ecules are linked *via* N—H⋯O=P hydrogen bonds to form extended chains along [010].

## Related literature

For a related structure, see: Pourayoubi *et al.* (2011 ▶).
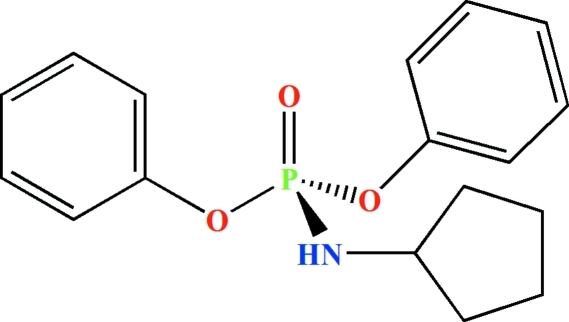


## Experimental

### 

#### Crystal data

C_17_H_20_NO_3_P
*M* *_r_* = 317.31
Monoclinic, 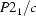

*a* = 18.0095 (4) Å
*b* = 5.3471 (1) Å
*c* = 17.9387 (4) Å
β = 109.731 (1)°
*V* = 1626.05 (6) Å^3^
*Z* = 4
Mo *K*α radiation
μ = 0.18 mm^−1^
*T* = 296 K
0.5 × 0.4 × 0.2 mm

#### Data collection

Bruker APEXII CCD diffractometer
Absorption correction: multi-scan (*SADABS*; Sheldrick, 2004 ▶) *T*_min_ = 0.709, *T*_max_ = 0.747
139394 measured reflections
3531 independent reflections
3180 reflections with *I* > 2σ(*I*)
*R*_int_ = 0.021

#### Refinement

*R*[*F* ^2^ > 2σ(*F* ^2^)] = 0.037
*wR*(*F* ^2^) = 0.110
*S* = 1.08
3531 reflections
240 parameters
H atoms treated by a mixture of independent and constrained refinement
Δρ_max_ = 0.23 e Å^−3^
Δρ_min_ = −0.28 e Å^−3^

### 

Data collection: *APEX2* (Bruker, 2005 ▶); cell refinement: *SAINT* (Bruker, 2005 ▶); data reduction: *SAINT*; program(s) used to solve structure: *SHELXS97* (Sheldrick, 2008 ▶); program(s) used to refine structure: *SHELXL97* (Sheldrick, 2008 ▶); molecular graphics: *Mercury* (Macrae *et al.*, 2008 ▶); software used to prepare material for publication: *SHELXTL* (Sheldrick, 2008 ▶) and *enCIFer* (Allen *et al.*, 2004 ▶).

## Supplementary Material

Crystal structure: contains datablocks I, global. DOI: 10.1107/S1600536811017028/lh5228sup1.cif

Structure factors: contains datablocks I. DOI: 10.1107/S1600536811017028/lh5228Isup2.hkl

Supplementary material file. DOI: 10.1107/S1600536811017028/lh5228Isup3.cml

Additional supplementary materials:  crystallographic information; 3D view; checkCIF report

## Figures and Tables

**Table 1:** Hydrogen-bond geometry (Å, °)

*D*—H⋯*A*	*D*—H	H⋯*A*	*D*⋯*A*	*D*—H⋯*A*
N—H⋯O1^i^	0.790 (19)	2.23 (2)	3.0039 (17)	167.7 (19)

Symmetry code: (i) 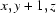.

**Fig. 1.**
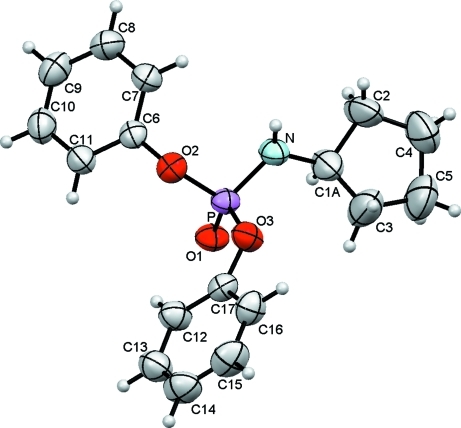
The molecular structure of the title compound with ellipsoids shown at the 50% probability level. The disorder is not shown.

## supplementary crystallographic information

### Comment

We have already studied the crystal structure of a diphenyl(amido)phosphonate, (C_6_H_5_O)_2_P(O)(NHCH_2_(2-ClC_6_H_4_) (Pourayoubi *et al.*, 2011). Here, we report the synthesis and crystal structure of title compound.

The P═O, P—O and P—N bond lengths are standard for (amido)phosphonate compounds. The P atom has a distorted tetrahedral configuration (Fig. 1) with the bond angles in the range of 99.72 (6)° [O2–P–O3] to 115.93 (6)° [O1–P–O2]. The phosphoryl group and the N–H unit are in an *anti* orientation with respect to each other which allows adjacent molecules to form extended chains along [010] *via* N—H···O(P) hydrogen bonds (Table 1).

### Experimental

To a solution of (C_6_H_5_O)_2_P(O)Cl in chloroform, a solution of cyclopentylamine (1:2 mole ratio) in chloroform was added at 273 K. After 4 h stirring, the solvent was removed and product was washed with distilled water. Single crystals were obtained from a solution of the title compound in CH_3_OH after slow evaporation at room temperature.

### Refinement

The nitrogen bonded hydrogen atom was found in a differnce Fourier map and allowed to refine while all other hydrogen atoms were placed in calculated positions with C–H = 0.93-0.98Å and with U_iso_(H) = 1.2U_eq_(C). The –CH_2_–CH_2_–CH_2_–CH_2_– sequence of atoms in the cyclopentyl ring are disordered over two sets of sites with refined occupancies 0.574 (10) and 0.426 (10).

### Crystal data

**Table:**

C_17_H_20_NO_3_P	*F*(000) = 672
*M**_r_* = 317.31	*D*_x_ = 1.296 Mg m^−^^3^
Monoclinic, *P*2_1_/*c*	Mo *K*α radiation, λ = 0.71073 Å
Hall symbol: -P 2ybc	Cell parameters from 9100 reflections
*a* = 18.0095 (4) Å	θ = 2.3–32.9°
*b* = 5.3471 (1) Å	µ = 0.18 mm^−^^1^
*c* = 17.9387 (4) Å	*T* = 296 K
β = 109.731 (1)°	Irregular, colorless
*V* = 1626.05 (6) Å^3^	0.5 × 0.4 × 0.2 mm
*Z* = 4	

### Data collection

**Table:**

Bruker APEXII CCD diffractometer	3531 independent reflections
Radiation source: fine-focus sealed tube	3180 reflections with *I* > 2σ(*I*)
graphite	*R*_int_ = 0.021
φ and ω scans	θ_max_ = 27.0°, θ_min_ = 2.4°
Absorption correction: multi-scan (*SADABS*; Sheldrick, 2004)	*h* = −23→23
*T*_min_ = 0.709, *T*_max_ = 0.747	*k* = −6→6
139394 measured reflections	*l* = −22→22

### Refinement

**Table:**

Refinement on *F*^2^	Primary atom site location: structure-invariant direct methods
Least-squares matrix: full	Secondary atom site location: difference Fourier map
*R*[*F*^2^ > 2σ(*F*^2^)] = 0.037	Hydrogen site location: inferred from neighbouring sites
*wR*(*F*^2^) = 0.110	H atoms treated by a mixture of independent and constrained refinement
*S* = 1.08	*w* = 1/[σ^2^(*F*_o_^2^) + (0.0497*P*)^2^ + 0.5717*P*] where *P* = (*F*_o_^2^ + 2*F*_c_^2^)/3
3531 reflections	(Δ/σ)_max_ = 0.04
240 parameters	Δρ_max_ = 0.23 e Å^−^^3^
0 restraints	Δρ_min_ = −0.28 e Å^−^^3^

### Special details

**Table:**

Geometry. All s.u.'s (except the s.u. in the dihedral angle between two l.s. planes) are estimated using the full covariance matrix. The cell s.u.'s are taken into account individually in the estimation of s.u.'s in distances, angles and torsion angles; correlations between s.u.'s in cell parameters are only used when they are defined by crystal symmetry. An approximate (isotropic) treatment of cell s.u.'s is used for estimating s.u.'s involving l.s. planes.
Refinement. Refinement of *F*^2^ against ALL reflections. The weighted *R*-factor *wR* and goodness of fit *S* are based on *F*^2^, conventional *R*-factors *R* are based on *F*, with *F* set to zero for negative *F*^2^. The threshold expression of *F*^2^ > 2σ(*F*^2^) is used only for calculating *R*-factors(gt) *etc*. and is not relevant to the choice of reflections for refinement. *R*-factors based on *F*^2^ are statistically about twice as large as those based on *F*, and *R*- factors based on ALL data will be even larger.

### Fractional atomic coordinates and isotropic or equivalent isotropic displacement parameters (Å^2^)

**Table:**

	*x*	*y*	*z*	*U*_iso_*/*U*_eq_	Occ. (<1)
C1	0.11088 (11)	0.7234 (4)	0.15643 (15)	0.0736 (6)	
H1A	0.1163	0.5834	0.1931	0.088*	0.574 (10)
C2	0.0736 (5)	0.9286 (12)	0.1911 (5)	0.108 (3)	0.574 (10)
H2A	0.0896	0.9112	0.2482	0.130*	0.574 (10)
H2B	0.0900	1.0919	0.1790	0.130*	0.574 (10)
C3	0.0542 (3)	0.6434 (17)	0.0887 (4)	0.105 (3)	0.574 (10)
H3A	0.0622	0.4685	0.0793	0.125*	0.574 (10)
H3B	0.0562	0.7400	0.0437	0.125*	0.574 (10)
C4	−0.0107 (7)	0.901 (2)	0.1558 (11)	0.114 (5)	0.574 (10)
H4A	−0.0339	1.0511	0.1267	0.136*	0.574 (10)
H4B	−0.0344	0.8714	0.1962	0.136*	0.574 (10)
C5	−0.0237 (3)	0.6791 (19)	0.1003 (5)	0.111 (2)	0.574 (10)
H5A	−0.0381	0.5316	0.1238	0.133*	0.574 (10)
H5B	−0.0650	0.7139	0.0503	0.133*	0.574 (10)
H1AA	0.1016	0.5426	0.1550	0.088*	0.426 (10)
C2A	0.0824 (7)	0.830 (4)	0.2078 (6)	0.164 (7)	0.426 (10)
H2A1	0.1020	1.0001	0.2192	0.197*	0.426 (10)
H2A2	0.0978	0.7367	0.2570	0.197*	0.426 (10)
C3A	0.0515 (4)	0.856 (3)	0.0735 (5)	0.121 (4)	0.426 (10)
H3A1	0.0694	1.0221	0.0664	0.146*	0.426 (10)
H3A2	0.0477	0.7548	0.0274	0.146*	0.426 (10)
C4A	−0.0101 (9)	0.830 (4)	0.1673 (13)	0.151 (8)	0.426 (10)
H4A1	−0.0320	0.6723	0.1766	0.181*	0.426 (10)
H4A2	−0.0335	0.9642	0.1882	0.181*	0.426 (10)
C5A	−0.0243 (6)	0.864 (3)	0.0878 (8)	0.131 (4)	0.426 (10)
H5A1	−0.0497	1.0243	0.0711	0.157*	0.426 (10)
H5A2	−0.0590	0.7336	0.0577	0.157*	0.426 (10)
C6	0.38086 (8)	0.6852 (3)	0.24407 (8)	0.0428 (3)	
C7	0.37482 (10)	0.8825 (3)	0.29063 (10)	0.0530 (4)	
H7	0.3370	1.0062	0.2706	0.064*	
C8	0.42607 (11)	0.8939 (4)	0.36780 (11)	0.0642 (4)	
H8	0.4225	1.0252	0.4004	0.077*	
C9	0.48253 (11)	0.7112 (4)	0.39663 (11)	0.0657 (5)	
H9	0.5167	0.7189	0.4487	0.079*	
C10	0.48839 (10)	0.5181 (4)	0.34858 (12)	0.0637 (4)	
H10	0.5271	0.3968	0.3680	0.076*	
C11	0.43704 (9)	0.5027 (3)	0.27138 (10)	0.0534 (4)	
H11	0.4405	0.3714	0.2387	0.064*	
C12	0.29427 (10)	0.1941 (3)	0.02869 (10)	0.0553 (4)	
H12	0.3133	0.1604	0.0827	0.066*	
C13	0.31354 (11)	0.0410 (4)	−0.02407 (12)	0.0640 (5)	
H13	0.3458	−0.0972	−0.0052	0.077*	
C14	0.28593 (12)	0.0892 (4)	−0.10368 (12)	0.0708 (5)	
H14	0.2993	−0.0153	−0.1386	0.085*	
C15	0.23842 (12)	0.2924 (4)	−0.13150 (10)	0.0706 (5)	
H15	0.2194	0.3250	−0.1856	0.085*	
C16	0.21843 (10)	0.4497 (4)	−0.08008 (9)	0.0569 (4)	
H16	0.1866	0.5886	−0.0991	0.068*	
C17	0.24637 (9)	0.3976 (3)	−0.00026 (8)	0.0452 (3)	
N	0.19186 (8)	0.7736 (3)	0.16096 (8)	0.0493 (3)	
O1	0.23902 (7)	0.31497 (19)	0.17324 (6)	0.0509 (3)	
O2	0.33107 (6)	0.6759 (2)	0.16438 (6)	0.0469 (3)	
O3	0.22198 (7)	0.5640 (2)	0.04688 (6)	0.0529 (3)	
P	0.24508 (2)	0.56130 (6)	0.14026 (2)	0.04116 (13)	
H	0.2031 (11)	0.915 (4)	0.1566 (11)	0.057 (5)*	

### Atomic displacement parameters (Å^2^)

**Table:**

	*U*^11^	*U*^22^	*U*^33^	*U*^12^	*U*^13^	*U*^23^
C1	0.0531 (10)	0.0536 (10)	0.1190 (17)	−0.0082 (8)	0.0356 (11)	−0.0161 (11)
C2	0.072 (4)	0.092 (4)	0.175 (8)	−0.012 (2)	0.060 (5)	−0.073 (4)
C3	0.058 (2)	0.141 (5)	0.107 (4)	−0.013 (3)	0.019 (2)	−0.056 (4)
C4	0.072 (5)	0.107 (5)	0.170 (12)	0.018 (4)	0.052 (6)	−0.029 (5)
C5	0.050 (2)	0.147 (6)	0.129 (5)	−0.012 (3)	0.022 (3)	−0.027 (5)
C1A	0.0531 (10)	0.0536 (10)	0.1190 (17)	−0.0082 (8)	0.0356 (11)	−0.0161 (11)
C2A	0.061 (4)	0.38 (2)	0.062 (3)	−0.018 (9)	0.034 (3)	−0.024 (8)
C3A	0.061 (3)	0.210 (12)	0.087 (4)	0.012 (5)	0.018 (3)	0.046 (6)
C4A	0.060 (7)	0.28 (2)	0.128 (10)	−0.052 (9)	0.058 (7)	−0.058 (13)
C5A	0.075 (5)	0.165 (11)	0.148 (8)	0.014 (6)	0.032 (5)	0.035 (9)
C6	0.0413 (7)	0.0400 (7)	0.0492 (7)	−0.0041 (6)	0.0180 (6)	0.0033 (6)
C7	0.0535 (8)	0.0436 (8)	0.0596 (9)	0.0028 (7)	0.0161 (7)	−0.0015 (7)
C8	0.0679 (11)	0.0583 (10)	0.0613 (10)	−0.0034 (8)	0.0153 (8)	−0.0124 (8)
C9	0.0566 (10)	0.0737 (12)	0.0567 (9)	−0.0057 (9)	0.0059 (8)	0.0023 (9)
C10	0.0478 (8)	0.0617 (10)	0.0736 (11)	0.0073 (8)	0.0101 (8)	0.0083 (9)
C11	0.0486 (8)	0.0469 (8)	0.0650 (10)	0.0025 (7)	0.0198 (7)	−0.0024 (7)
C12	0.0640 (9)	0.0521 (9)	0.0489 (8)	0.0033 (7)	0.0180 (7)	−0.0015 (7)
C13	0.0666 (11)	0.0576 (10)	0.0727 (11)	0.0035 (8)	0.0300 (9)	−0.0089 (8)
C14	0.0743 (12)	0.0815 (14)	0.0667 (11)	−0.0092 (10)	0.0370 (10)	−0.0217 (10)
C15	0.0744 (12)	0.0967 (15)	0.0435 (8)	−0.0090 (11)	0.0238 (8)	−0.0067 (9)
C16	0.0545 (9)	0.0676 (11)	0.0463 (8)	−0.0004 (8)	0.0138 (7)	0.0055 (7)
C17	0.0462 (7)	0.0477 (8)	0.0410 (7)	−0.0069 (6)	0.0138 (6)	−0.0034 (6)
N	0.0497 (7)	0.0374 (7)	0.0636 (8)	−0.0043 (5)	0.0227 (6)	−0.0056 (6)
O1	0.0638 (6)	0.0359 (5)	0.0532 (6)	−0.0033 (5)	0.0199 (5)	0.0019 (4)
O2	0.0486 (6)	0.0486 (6)	0.0459 (5)	−0.0031 (4)	0.0190 (4)	0.0027 (4)
O3	0.0659 (7)	0.0478 (6)	0.0416 (5)	0.0109 (5)	0.0136 (5)	0.0021 (4)
P	0.0474 (2)	0.0344 (2)	0.0416 (2)	−0.00097 (14)	0.01477 (15)	0.00036 (13)

### Geometric parameters (Å, °)

**Table:**

C1—C3	1.365 (5)	C6—O2	1.4088 (17)
C1—N	1.458 (2)	C7—C8	1.382 (2)
C1—C2	1.524 (6)	C7—H7	0.9300
C1—H1A	0.9800	C8—C9	1.378 (3)
C2—C4	1.442 (16)	C8—H8	0.9300
C2—H2A	0.9700	C9—C10	1.372 (3)
C2—H2B	0.9700	C9—H9	0.9300
C3—C5	1.499 (7)	C10—C11	1.384 (2)
C3—H3A	0.9700	C10—H10	0.9300
C3—H3B	0.9700	C11—H11	0.9300
C4—C5	1.516 (16)	C12—C17	1.377 (2)
C4—H4A	0.9700	C12—C13	1.381 (2)
C4—H4B	0.9700	C12—H12	0.9300
C5—H5A	0.9700	C13—C14	1.369 (3)
C5—H5B	0.9700	C13—H13	0.9300
C2A—C4A	1.577 (19)	C14—C15	1.369 (3)
C2A—H2A1	0.9700	C14—H14	0.9300
C2A—H2A2	0.9700	C15—C16	1.383 (3)
C3A—C5A	1.472 (12)	C15—H15	0.9300
C3A—H3A1	0.9700	C16—C17	1.376 (2)
C3A—H3A2	0.9700	C16—H16	0.9300
C4A—C5A	1.37 (3)	C17—O3	1.3968 (18)
C4A—H4A1	0.9700	N—P	1.6078 (14)
C4A—H4A2	0.9700	N—H	0.793 (19)
C5A—H5A1	0.9700	O1—P	1.4630 (11)
C5A—H5A2	0.9700	O2—P	1.5839 (10)
C6—C11	1.372 (2)	O3—P	1.5838 (11)
C6—C7	1.373 (2)		
			
C3—C1—N	122.8 (3)	C11—C6—C7	122.00 (15)
C3—C1—C2	106.8 (4)	C11—C6—O2	118.51 (13)
N—C1—C2	114.5 (3)	C7—C6—O2	119.39 (13)
C3—C1—H1A	103.5	C6—C7—C8	118.69 (15)
N—C1—H1A	103.5	C6—C7—H7	120.7
C2—C1—H1A	103.5	C8—C7—H7	120.7
C4—C2—C1	106.9 (6)	C9—C8—C7	120.23 (17)
C4—C2—H2A	110.3	C9—C8—H8	119.9
C1—C2—H2A	110.4	C7—C8—H8	119.9
C4—C2—H2B	110.3	C10—C9—C8	120.07 (17)
C1—C2—H2B	110.3	C10—C9—H9	120.0
H2A—C2—H2B	108.6	C8—C9—H9	120.0
C1—C3—C5	106.9 (4)	C9—C10—C11	120.43 (17)
C1—C3—H3A	110.3	C9—C10—H10	119.8
C5—C3—H3A	110.3	C11—C10—H10	119.8
C1—C3—H3B	110.3	C6—C11—C10	118.57 (16)
C5—C3—H3B	110.3	C6—C11—H11	120.7
H3A—C3—H3B	108.6	C10—C11—H11	120.7
C2—C4—C5	105.9 (6)	C17—C12—C13	118.71 (16)
C2—C4—H4A	110.6	C17—C12—H12	120.6
C5—C4—H4A	110.5	C13—C12—H12	120.6
C2—C4—H4B	110.5	C14—C13—C12	121.09 (18)
C5—C4—H4B	110.6	C14—C13—H13	119.5
H4A—C4—H4B	108.7	C12—C13—H13	119.5
C3—C5—C4	104.2 (6)	C13—C14—C15	119.48 (17)
C3—C5—H5A	110.9	C13—C14—H14	120.3
C4—C5—H5A	110.9	C15—C14—H14	120.3
C3—C5—H5B	110.9	C14—C15—C16	120.75 (17)
C4—C5—H5B	110.9	C14—C15—H15	119.6
H5A—C5—H5B	108.9	C16—C15—H15	119.6
C4A—C2A—H2A1	110.5	C17—C16—C15	118.99 (18)
C4A—C2A—H2A2	110.6	C17—C16—H16	120.5
H2A1—C2A—H2A2	108.7	C15—C16—H16	120.5
C5A—C3A—H3A1	111.4	C12—C17—C16	120.99 (15)
C5A—C3A—H3A2	111.3	C12—C17—O3	124.08 (13)
H3A1—C3A—H3A2	109.2	C16—C17—O3	114.93 (14)
C5A—C4A—C2A	106.1 (12)	C1—N—P	121.36 (12)
C5A—C4A—H4A1	110.5	C1—N—H	117.0 (14)
C2A—C4A—H4A1	110.5	P—N—H	117.3 (14)
C5A—C4A—H4A2	110.5	C6—O2—P	121.30 (8)
C2A—C4A—H4A2	110.5	C17—O3—P	127.62 (10)
H4A1—C4A—H4A2	108.7	O1—P—O2	115.93 (6)
C4A—C5A—C3A	108.6 (9)	O1—P—O3	114.03 (6)
C4A—C5A—H5A1	110.0	O2—P—O3	99.72 (6)
C3A—C5A—H5A1	109.9	O1—P—N	114.24 (7)
C4A—C5A—H5A2	110.0	O2—P—N	105.55 (6)
C3A—C5A—H5A2	110.0	O3—P—N	105.88 (7)
H5A1—C5A—H5A2	108.4		
			
C3—C1—C2—C4	18.7 (10)	C13—C12—C17—C16	−0.5 (2)
N—C1—C2—C4	158.1 (8)	C13—C12—C17—O3	179.15 (15)
N—C1—C3—C5	−165.1 (4)	C15—C16—C17—C12	0.8 (3)
C2—C1—C3—C5	−29.9 (7)	C15—C16—C17—O3	−178.89 (15)
C1—C2—C4—C5	0.3 (13)	C3—C1—N—P	−57.8 (5)
C1—C3—C5—C4	29.8 (11)	C2—C1—N—P	170.1 (4)
C2—C4—C5—C3	−17.2 (14)	C11—C6—O2—P	98.41 (14)
C2A—C4A—C5A—C3A	7(2)	C7—C6—O2—P	−85.20 (15)
C11—C6—C7—C8	−1.3 (2)	C12—C17—O3—P	2.0 (2)
O2—C6—C7—C8	−177.58 (15)	C16—C17—O3—P	−178.33 (11)
C6—C7—C8—C9	0.7 (3)	C6—O2—P—O1	−49.72 (12)
C7—C8—C9—C10	0.4 (3)	C6—O2—P—O3	−172.58 (10)
C8—C9—C10—C11	−1.1 (3)	C6—O2—P—N	77.80 (12)
C7—C6—C11—C10	0.7 (2)	C17—O3—P—O1	−46.75 (15)
O2—C6—C11—C10	177.00 (14)	C17—O3—P—O2	77.45 (13)
C9—C10—C11—C6	0.5 (3)	C17—O3—P—N	−173.19 (12)
C17—C12—C13—C14	0.1 (3)	C1—N—P—O1	−43.33 (18)
C12—C13—C14—C15	0.0 (3)	C1—N—P—O2	−171.87 (15)
C13—C14—C15—C16	0.3 (3)	C1—N—P—O3	82.99 (16)
C14—C15—C16—C17	−0.7 (3)		

### Hydrogen-bond geometry (Å, °)

**Table:**

*D*—H···*A*	*D*—H	H···*A*	*D*···*A*	*D*—H···*A*
N—H···O1^i^	0.790 (19)	2.23 (2)	3.0039 (17)	167.7 (19)

Symmetry codes: (i) *x*, *y*+1, *z*.
